# Pulmonary Tuberculosis Mimicking Metastatic Disease in a Patient With Two Previous Primary Malignancies: A Thoracoscopic Diagnostic Challenge

**DOI:** 10.7759/cureus.115084

**Published:** 2026-08-24

**Authors:** Ivan Bivolarski, Martin Dimitrov, Tanio Stefanov, Presian Stefanov, Stanislav Marangozov

**Affiliations:** 1 Chronotherapy and Cancer Research Unit, Integrated Oncology Centre Burgas, Burgas, BGR; 2 General, Vascular, and Visceral Surgery Department, Kreiskrankenhaus Greiz, Greiz, DEU; 3 Surgical Oncology Department, Burgasmed LTD, Burgas, BGR; 4 Thoracic Surgery Department, Medical University of Plovdiv, Plovdiv, BGR

**Keywords:** breast cancer pathology, differential diagnosis, mediastinal lymphadenopathy, pulmonary nodule, pulmonary tuberculosis, renal cell carcinoma, thoracic surgery, thoracoscopic biopsy, vats, wedge resection

## Abstract

Pulmonary nodules detected in patients with a history of multiple malignancies are frequently presumed to represent metastatic disease. However, infectious granulomatous disorders, particularly pulmonary tuberculosis, may closely mimic thoracic metastases and create substantial diagnostic challenges. We present the case of a 64-year-old woman with a history of surgically treated renal cell carcinoma and breast cancer who underwent routine thoracic imaging that revealed a pulmonary nodule accompanied by mediastinal lymphadenopathy. Given her oncological history, metastatic disease was considered an important diagnostic possibility. The patient underwent video-assisted thoracoscopic surgery (VATS), including wedge resection of the pulmonary lesion and mediastinal lymph node biopsy. Histopathological examination demonstrated necrotizing granulomatous inflammation composed of epithelioid histiocytes and multinucleated giant cells, without evidence of malignancy. These findings favored an infectious granulomatous process, particularly pulmonary tuberculosis. The patient was subsequently referred to a specialized tuberculosis center for microbiological evaluation and further management. This case highlights the importance of including pulmonary tuberculosis in the differential diagnosis of newly detected pulmonary lesions, even in patients with multiple previous malignancies. It also underscores the limitations of imaging alone and reinforces the essential role of tissue diagnosis in preventing inappropriate cancer staging and unnecessary oncological treatment while facilitating timely disease-specific evaluation and management.

## Introduction

Pulmonary nodules and intrathoracic lymphadenopathy in patients with a history of malignancy are frequently interpreted as manifestations of recurrent or metastatic disease. This assumption is particularly common in patients previously treated for breast cancer or renal cell carcinoma, both of which have a recognized propensity for pulmonary metastasis. Consequently, a thoracic lesion first identified during surveillance of a cancer survivor often prompts extensive oncological evaluation and may substantially influence staging and treatment decisions [1]. An incorrect presumption of metastatic disease may expose the patient to unnecessary investigations, psychological distress, and potentially inappropriate oncological treatment.

Despite major advances in thoracic imaging, differentiating malignant lesions from infectious or inflammatory processes remains challenging. Computed tomography (CT) and positron emission tomography (PET) findings may overlap considerably between metastatic tumors and granulomatous diseases. Pulmonary tuberculosis is among the most important non-malignant conditions capable of mimicking primary lung cancer, metastatic disease, or cancer recurrence, particularly when it presents as a solitary pulmonary nodule, pulmonary mass, or mediastinal lymphadenopathy. In such circumstances, radiological findings alone are often insufficient to establish a definitive diagnosis [2,3].

A previous oncological history further complicates the diagnostic process by increasing the pre-test probability of recurrent or metastatic disease. Consequently, pulmonary tuberculosis may be overlooked, particularly in patients without constitutional symptoms or an evident epidemiological risk. Moreover, fluorine-18 fluorodeoxyglucose positron emission tomography/computed tomography (^18^F-FDG PET/CT) may demonstrate intense metabolic activity in both malignancy and active granulomatous inflammation, thereby increasing diagnostic uncertainty [1,2,4].

The differential diagnosis of granulomatous thoracic disease also includes sarcoidosis, which may resemble tuberculosis clinically and radiologically. Histopathological overlap may further complicate distinction between these conditions; therefore, tissue morphology should be interpreted together with epidemiological information, imaging findings, and microbiological or molecular investigations whenever available. Emerging approaches based on immunological profiling and machine learning models may assist this distinction in the future, although they are not yet established components of routine diagnostic practice [5].

Histopathological examination remains central to establishing a tissue diagnosis when imaging findings are inconclusive. Video-assisted thoracoscopic surgery (VATS) offers a minimally invasive approach for obtaining adequate tissue samples and plays a pivotal role in distinguishing malignancy from infectious or inflammatory disease. Identification of necrotizing granulomatous inflammation may fundamentally alter clinical management by redirecting the diagnostic pathway from presumed metastatic disease toward appropriate microbiological assessment and infectious disease care. Current thoracic surgical recommendations continue to emphasize tissue diagnosis for indeterminate pulmonary nodules when non-invasive investigations fail to provide a definitive diagnosis [6,7]. Effective communication among clinicians, radiologists, thoracic surgeons, pathologists, and microbiologists is essential for integrating these findings and reaching an accurate diagnosis.

Tuberculosis remains a major global health problem, with millions of new cases diagnosed annually. Although pulmonary tuberculosis commonly presents with recognizable clinical and radiological features, atypical manifestations continue to pose important diagnostic challenges, particularly in patients with a history of malignancy [8].

The present case is noteworthy because several independent clinical features strongly suggested metastatic disease: two previous primary malignancies, recurrence of one of these cancers, a pulmonary nodule, and mediastinal lymphadenopathy. The thoracic abnormalities were first identified on the current CT examination; this wording does not imply that their precise time of onset was known. Despite the highly suspicious oncological context, histopathological examination of tissue obtained by VATS demonstrated necrotizing granulomatous inflammation without evidence of malignancy, redirecting the diagnostic evaluation toward pulmonary tuberculosis. This case illustrates the importance of maintaining a broad differential diagnosis and obtaining tissue confirmation before classifying a thoracic lesion as recurrent or metastatic cancer or initiating oncological treatment.

## Case presentation

In May 2023, a 64-year-old woman was referred to the Department of Thoracic Surgery for diagnostic evaluation of a pulmonary lesion identified during routine oncological surveillance. Her medical history was notable for two previous primary malignancies, insulin-dependent diabetes mellitus, and thalassemia.

In 2009, she underwent right nephrectomy for clear cell renal cell carcinoma. During subsequent surveillance, fluorine-18 fluorodeoxyglucose positron emission tomography/computed tomography (^18^F-FDG PET/CT) performed in January 2018 demonstrated metabolically active pretracheal, paratracheal, subcarinal, and bilateral hilar lymphadenopathy. The lymph nodes measured up to 30 mm, and many contained extensive calcifications. Their characteristic mediastinal distribution formed a reported “lambda sign,” which was interpreted as suggestive of sarcoidosis. Several bilateral pleural-based pulmonary nodules measuring up to 5 mm were also identified but were too small for reliable metabolic characterization. No other metabolically active lesions suspicious for recurrent or disseminated malignancy were detected.

Because of the prominent mediastinal lymphadenopathy, surgical tissue sampling was attempted in May 2018. Cervical exploration was performed; however, no representative enlarged lymph node could be identified. Histopathological examination yielded only fibroadipose tissue with hemorrhage and a limited inflammatory infiltrate, without identifiable lymph node tissue or evidence of malignant infiltration. The procedure was therefore considered non-diagnostic, and imaging surveillance was continued.

Follow-up ^18^F-FDG PET/CT performed in 2021 demonstrated persistent metabolically active, partially calcified bilateral hilar and mediastinal lymphadenopathy without appreciable interval change. The bilateral subpleural micronodules also remained morphologically stable and metabolically inactive. No evidence of recurrent or disseminated renal cell carcinoma was identified.

In July 2022, the mediastinal lymph nodes remained metabolically active, with a maximum standardized uptake value (SUVmax) of up to 10.08, but showed no significant morphological progression. The bilateral pulmonary nodules also remained stable. In contrast, a lesion in the upper medial quadrant of the left breast increased from approximately 9 × 10 mm to 10.1 × 11.3 mm, while its SUVmax increased from 2.52 to 5.81. A mildly FDG-avid left axillary lymph node was also detected.

In January 2023, a Tru-cut biopsy of the left breast lesion demonstrated grade 2 invasive ductal carcinoma. Immunohistochemical analysis showed strong estrogen receptor expression (Allred score: 8/8), low progesterone receptor expression (Allred score: 2/8), human epidermal growth factor receptor 2 (HER2) overexpression (3+), and a Ki-67 proliferation index of 30% [9]. The disease was clinically staged as cT1N1M0 (stage IIA). PET/CT performed later that month demonstrated further morphological and metabolic progression of the breast lesion, which measured 16.4 × 10.5 mm and had an SUVmax of 10.0. The ipsilateral axillary lymph node remained mildly FDG-avid, with an SUVmax of 2.74. In contrast, the mediastinal lymphadenopathy had remained essentially stable since 2018 and was again interpreted radiologically as most likely representing sarcoidosis. Six bilateral pulmonary micronodules remained metabolically inactive and morphologically stable.

The patient received neoadjuvant systemic therapy with intravenous docetaxel at a total dose of 120 mg per cycle, intravenous pertuzumab at a loading dose of 840 mg followed by 420 mg per cycle, and subcutaneous trastuzumab at a fixed dose of 600 mg per cycle. Treatment was administered every three weeks for a total of eight cycles. She subsequently underwent left breast-conserving surgery and ipsilateral axillary lymph node excision in May 2023. Histopathological examination showed no residual invasive carcinoma, with only an in situ component remaining and no metastatic involvement of the examined axillary lymph nodes (ypTisN0M0). The resection margins were free of disease. Adjuvant radiotherapy to the left breast was completed in September 2023, with a total dose of 50 Gy delivered in 25 daily fractions of 2 Gy. Adjuvant endocrine therapy with oral letrozole at a dose of 2.5 mg once daily was subsequently initiated.

Follow-up PET/CT in January 2024 showed no metabolically active breast lesion or axillary lymphadenopathy. The longstanding mediastinal lymphadenopathy persisted, although its metabolic activity had decreased, with an SUVmax of 5.86 compared with 7.40 on the preceding examination. Bilateral pulmonary micronodules remained visible. Pleural thickening within the irradiated region of the left hemithorax demonstrated increased FDG uptake, with an SUVmax of 5.27, while several micronodules in the basal segments of the right lung showed newly apparent metabolic activity, with an SUVmax of up to 4.26. An inflammatory etiology could not be excluded.

**Figure 1:**
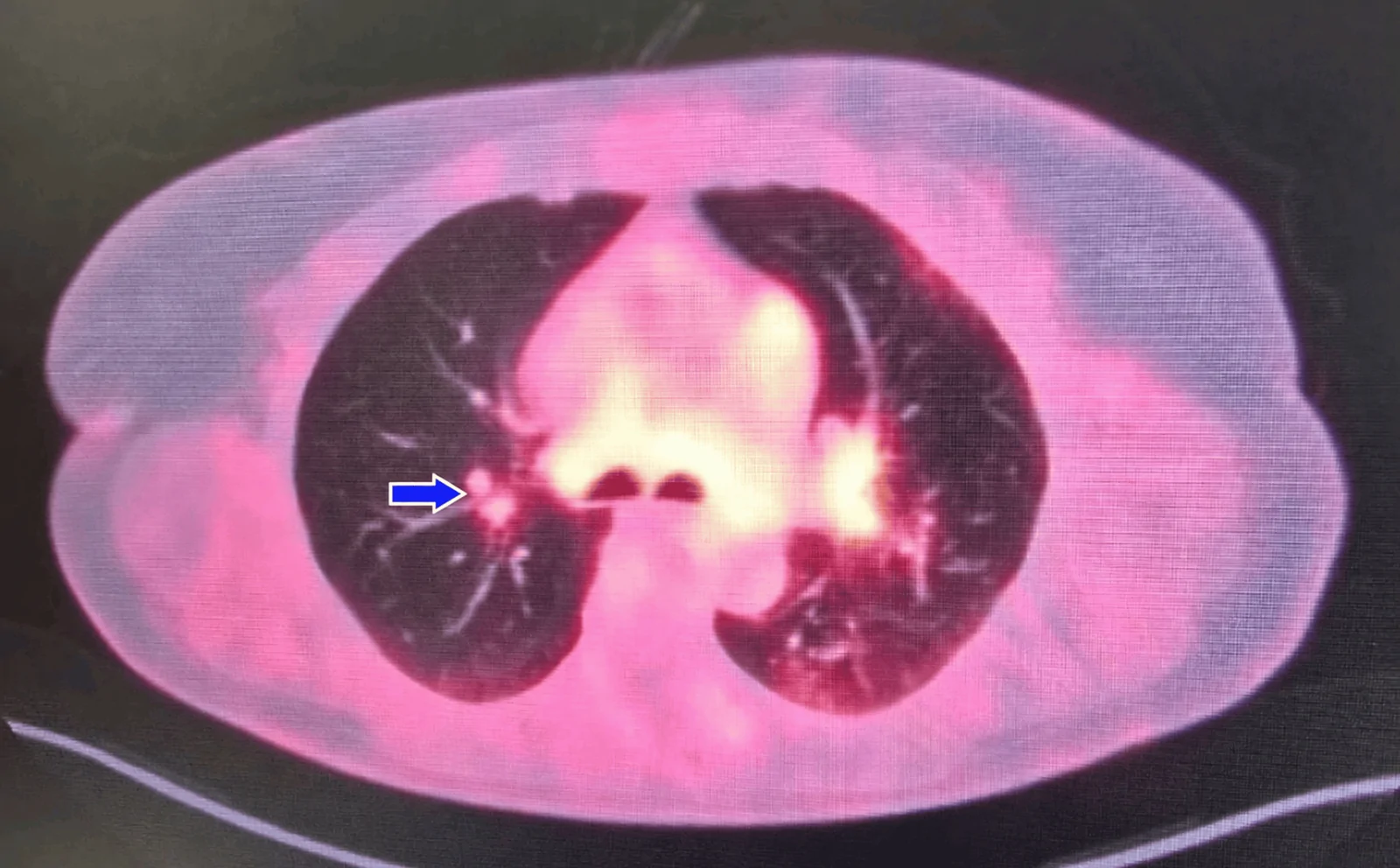
Preoperative axial fused 18F-FDG PET/CT image Axial ^18^F-FDG PET/CT image demonstrating increased metabolic activity in the bilateral hilar and mediastinal lymph nodes. The blue arrow indicates an FDG-avid right hilar lymph node. ^18^F-FDG PET/CT: fluorine-18 fluorodeoxyglucose positron emission tomography/computed tomography

A subsequent PET/CT examination in June 2024 demonstrated no substantial interval change. Metabolically active mediastinal lymph nodes persisted, with an SUVmax of up to 6.84. The left pulmonary micronodules remained metabolically inactive, and the previously FDG-avid pleural thickening no longer demonstrated increased uptake. A basal right pulmonary nodule remained mildly FDG-avid, although its SUVmax had decreased from 4.26 to 3.16. No new metabolically active lesions or evidence of local recurrence of either malignancy was identified (Figure 1).

**Figure 2:**
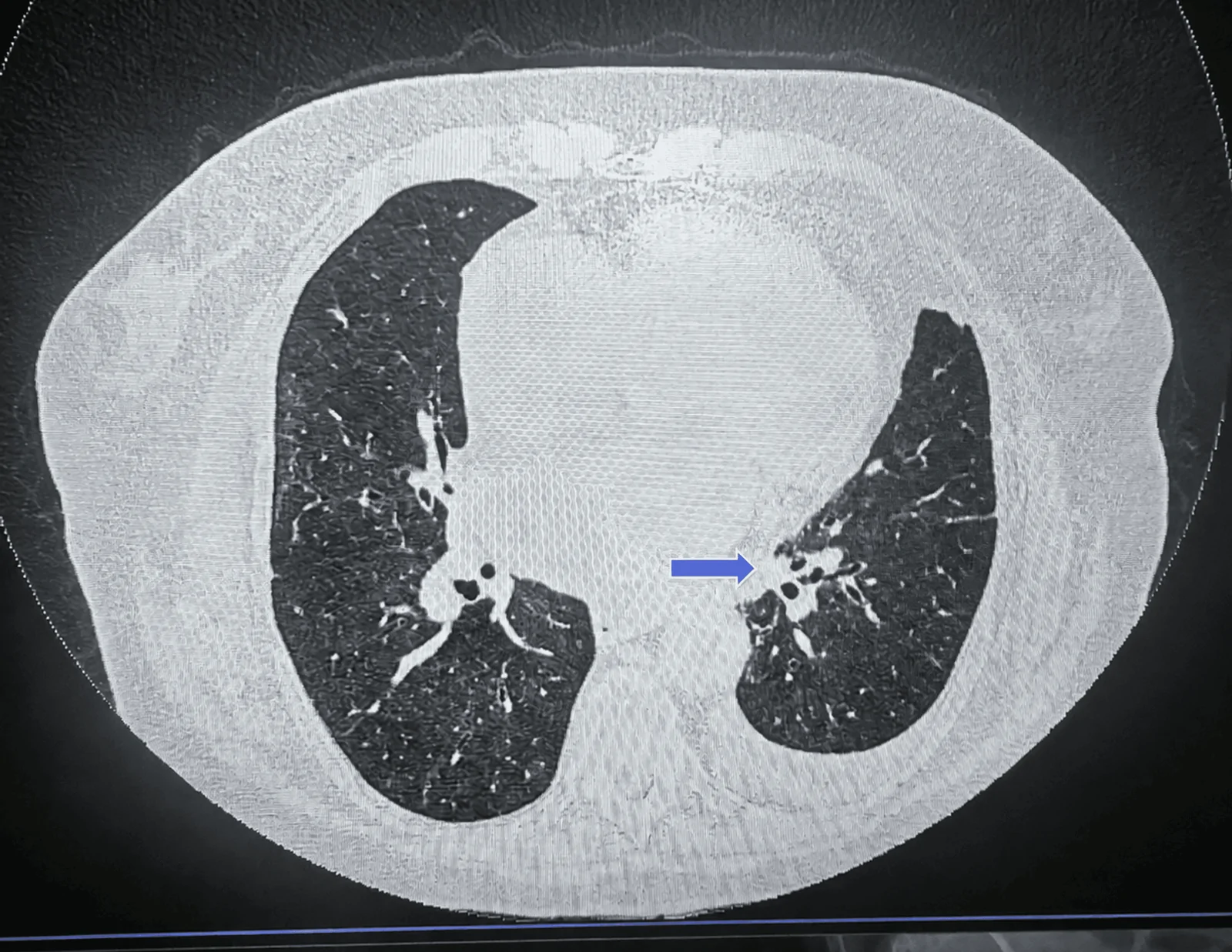
Axial chest CT image demonstrating left hilar lymphadenopathy The blue arrow indicates the enlarged left hilar lymph nodes. CT: computed tomography

During subsequent surveillance, contrast-enhanced chest CT demonstrated a well-circumscribed pulmonary nodule measuring approximately 0.8 × 1.3 cm in the left lung, accompanied by ipsilateral hilar and mediastinal lymphadenopathy (Figure 2). Despite the longstanding granulomatous-appearing thoracic abnormalities, the development of a dominant pulmonary lesion in a patient with two previous primary malignancies raised renewed concern for metastatic disease. Imaging findings alone were considered insufficient to distinguish malignancy from an inflammatory or granulomatous process.

At presentation, the patient was in good general condition. She denied fever, unexplained weight loss, night sweats, chronic cough, hemoptysis, or known exposure to tuberculosis. Her vital signs were stable, and no palpable peripheral lymphadenopathy was identified. Pulmonary auscultation demonstrated mildly diminished breath sounds bilaterally, without signs of respiratory distress.

Following multidisciplinary discussion, the patient underwent video-assisted thoracoscopic surgery (VATS) to obtain a definitive tissue diagnosis. Intraoperatively, the pulmonary lesion and enlarged mediastinal lymph nodes were identified. Thoracoscopic wedge resection of the pulmonary nodule and biopsy of the mediastinal lymph nodes were performed. Pleural scarification was also carried out, and two thoracic drains were placed at the conclusion of the procedure.

**Figure 3:**
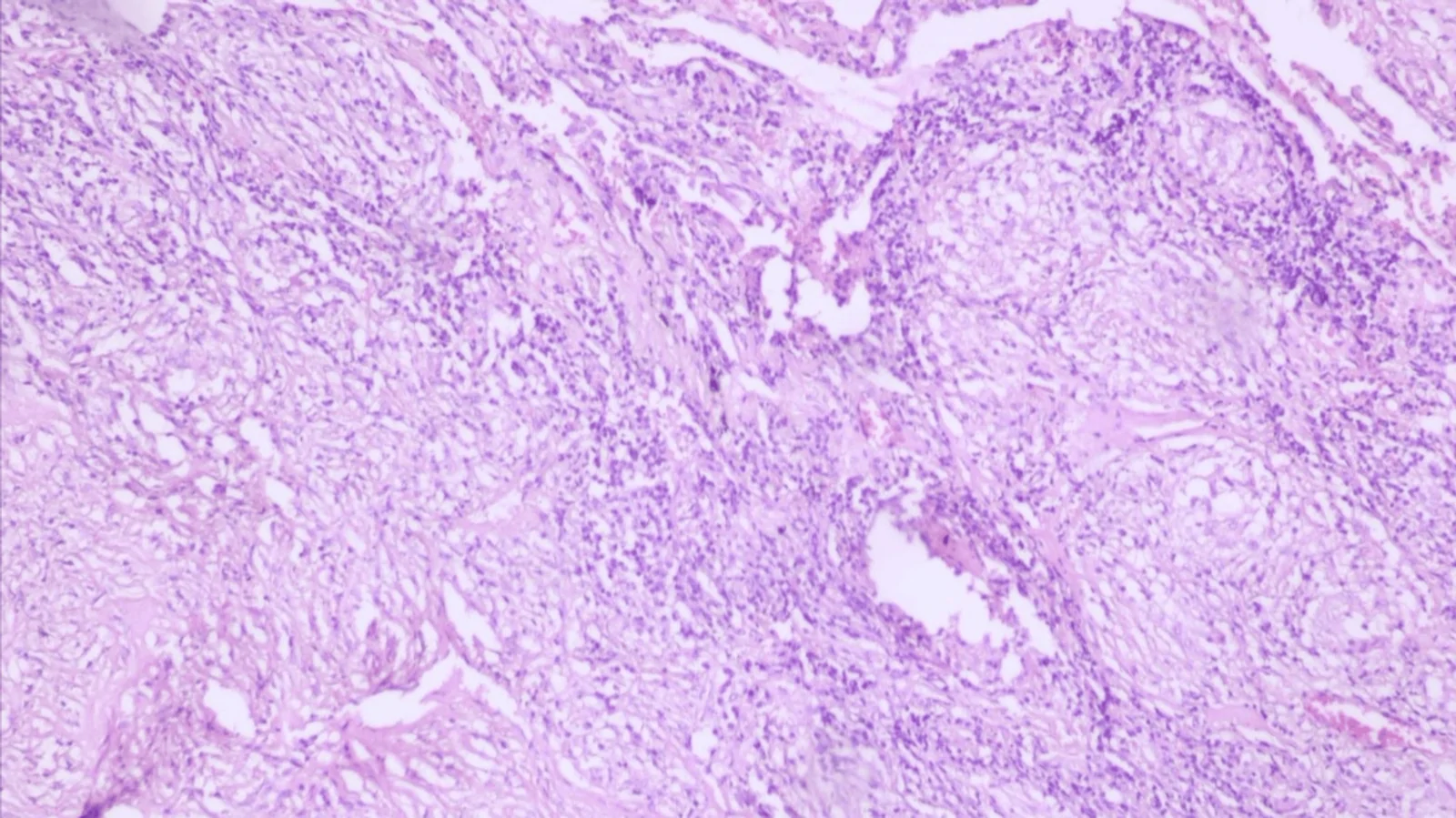
Histopathological appearance of granulomatous inflammation Hematoxylin and eosin-stained section showing multiple well-formed granulomas composed predominantly of epithelioid histiocytes. No malignant cells are identified in the illustrated field.

Histopathological examination demonstrated extensive necrotizing granulomatous inflammation composed of epithelioid histiocytes and multinucleated giant cells (Figure 3). No evidence of metastatic renal cell carcinoma, metastatic breast carcinoma, or primary pulmonary malignancy was identified. The morphological findings favored an infectious granulomatous process, particularly tuberculosis, while other infectious and non-infectious causes of necrotizing granulomatous inflammation remained within the differential diagnosis.

The postoperative course was uneventful. The thoracic drains were removed after satisfactory re-expansion of the lung, and the patient remained afebrile and hemodynamically stable throughout hospitalization. She was discharged in good clinical condition and referred to a specialized tuberculosis center for microbiological evaluation and further management.

## Discussion

Pulmonary lesions identified in patients with a history of malignancy remain a frequent diagnostic challenge in contemporary clinical practice. In such patients, pulmonary nodules are commonly presumed to represent recurrent or metastatic disease, particularly when the previous primary tumors have a recognized propensity for pulmonary dissemination. Both breast cancer and renal cell carcinoma frequently metastasize to the lungs, making metastatic disease an important initial diagnostic consideration in our patient. Consequently, alternative diagnoses may receive less attention during the initial evaluation, potentially delaying recognition of non-malignant conditions [1].

Tuberculosis remains a major global health problem despite substantial advances in diagnostic methods and treatment. According to the World Health Organization, millions of new cases continue to occur annually, with pulmonary tuberculosis representing the most common clinical form. Tuberculosis has long been described as “the great imitator” because it may resemble a broad spectrum of infectious, inflammatory, and neoplastic diseases, thereby creating considerable diagnostic uncertainty [6].

Radiological differentiation between pulmonary tuberculosis and malignancy can be particularly difficult. Both conditions may manifest as solitary pulmonary nodules, pulmonary masses, cavitary lesions, mediastinal lymphadenopathy, pleural abnormalities, or increased metabolic activity on fluorine-18 fluorodeoxyglucose positron emission tomography/computed tomography (^18^F-FDG PET/CT). No single imaging characteristic reliably distinguishes tuberculosis from malignant disease in every clinical setting [7-11]. Moreover, granulomatous inflammation may demonstrate intense FDG uptake, producing metabolic findings that are virtually indistinguishable from those of malignant tumors [4,11]. Imaging findings alone may therefore be insufficient to establish a definitive diagnosis.

This diagnostic challenge becomes even more pronounced in patients with a history of cancer. Several case reports have described pulmonary tuberculosis presenting as a suspicious pulmonary mass or presumed metastatic lesion, leading initially to consideration of primary lung cancer or recurrent metastatic disease [2,3,12]. In the present case, the diagnostic complexity was increased by the presence of longstanding metabolically active mediastinal and hilar lymphadenopathy, initially interpreted as sarcoidosis, together with pulmonary nodules that subsequently demonstrated metabolic activity.

A previous oncological history may also be relevant to the risk of tuberculosis. Dobler et al. reported that patients with solid malignancies have a higher risk of developing tuberculosis than the general population. Advanced age, previous anticancer treatment, and associated comorbidities may contribute to immune dysfunction and increased susceptibility to mycobacterial infection [1]. Our patient had two distinct primary malignancies and insulin-dependent diabetes mellitus, the latter being an established risk factor for tuberculosis and a potential contributor to atypical disease presentation [7].

The clinical suspicion of metastatic disease in this case was reinforced by several independent factors: two previous primary malignancies, recently treated breast cancer, a pulmonary nodule with newly apparent metabolic activity, and persistent mediastinal lymphadenopathy. Together, these findings created a compelling radiological impression of recurrent or metastatic disease and illustrate how a high pre-test probability may influence diagnostic reasoning in oncology. Nevertheless, the pulmonary abnormality was not assumed to be anatomically new: the relevant change was the development of FDG avidity in a previously observed nodule, and its exact biological time of onset could not be determined.

Histopathological evaluation remains central when imaging findings are inconclusive. Although contemporary imaging provides valuable anatomical and functional information, tissue diagnosis is essential before major therapeutic decisions are made whenever a representative specimen can be obtained safely. Current thoracic surgical recommendations support histological confirmation of indeterminate pulmonary nodules when non-invasive investigations fail to establish a definitive diagnosis [5].

In our patient, thoracoscopic wedge resection and mediastinal lymph node biopsy provided adequate tissue for pathological evaluation. Histological examination demonstrated necrotizing granulomatous inflammation composed of epithelioid histiocytes and multinucleated giant cells, without evidence of metastatic carcinoma or primary lung malignancy. These findings fundamentally altered the diagnostic pathway by redirecting clinical management from presumed recurrent malignancy toward evaluation for granulomatous infection. Similar diagnostic dilemmas have been reported in pulmonary and extrapulmonary tuberculosis, in which tissue sampling proved essential for excluding malignancy and guiding further investigation [12,13].

Histopathological findings should not, however, be interpreted in isolation. Necrotizing granulomatous inflammation strongly raises suspicion of an infectious etiology, including tuberculosis, but it is not independently specific for *Mycobacterium tuberculosis*. Accurate etiological diagnosis requires integration of the clinical history, epidemiological risk, radiological findings, histomorphology, and microbiological or molecular investigations, including acid-fast staining, mycobacterial culture, and nucleic acid amplification testing whenever available. Close communication among clinicians, radiologists, thoracic surgeons, pathologists, and microbiologists is therefore essential for establishing the diagnosis and avoiding inappropriate treatment.

The distinction between tuberculosis and sarcoidosis was particularly relevant in this case. Both conditions may present with mediastinal and hilar lymphadenopathy, pulmonary nodules, increased FDG uptake, and granulomatous inflammation. The longstanding, partially calcified lymphadenopathy with a reported lambda sign distribution and minimal morphological progression supported the initial radiological interpretation of sarcoidosis. Conversely, the subsequent finding of necrotizing granulomatous inflammation raised concern for tuberculosis and prompted specialized pulmonary and microbiological evaluation. Because substantial clinical and radiological overlap exists, neither imaging nor granuloma morphology should be used as the sole discriminator. Emerging methods based on immunological profiling and machine learning models may improve differentiation between tuberculosis and sarcoidosis, although they are not yet established for routine clinical use [13].

Had the diagnosis been based solely on the oncological history and radiological findings, the patient might have been incorrectly classified as having recurrent metastatic cancer, potentially resulting in inappropriate staging and unnecessary systemic treatment. Obtaining tissue was therefore clinically important not only for diagnostic accuracy but also for preventing the physical and psychological burden associated with an incorrect diagnosis of metastatic disease.

The coexistence of malignancy and active or latent tuberculosis may also affect contemporary immunotherapy decisions. Programmed death-ligand 1 (PD-L1) expression is used as a predictive biomarker in several malignancies; however, infection-associated immune activation may complicate its biological interpretation. Furthermore, immune checkpoint inhibition can modify host-mycobacterial immune interactions and has been associated with the development or reactivation of tuberculosis. Active infection should therefore be recognized and appropriately managed before immune checkpoint inhibitor therapy is initiated. Treatment decisions should be based on multidisciplinary assessment rather than PD-L1 expression alone, and additional research is required to define reliable biomarkers and optimal management strategies for patients with concomitant cancer and tuberculosis [12,13].

This case reinforces an important principle in contemporary oncological practice: even highly suspicious radiological findings should not replace histopathological confirmation when representative tissue can be obtained safely. A broad differential diagnosis should be maintained in patients with previous malignancies because tuberculosis, sarcoidosis, and other granulomatous diseases may closely mimic recurrent or metastatic cancer. Timely tissue sampling, followed by integrated pathological and microbiological evaluation, can prevent inappropriate cancer staging and unnecessary oncological treatment while facilitating appropriate disease-specific management.

## Conclusions

Pulmonary tuberculosis can closely mimic recurrent metastatic disease, particularly in patients with a previous history of malignancy. This case illustrates how a combination of two prior primary cancers, a newly detected pulmonary nodule, and mediastinal lymphadenopathy created a compelling clinical impression of metastatic disease that was ultimately disproven by histopathological examination. The case reinforces the fundamental principle that tissue diagnosis should precede major therapeutic decisions whenever feasible. Early histopathological confirmation not only prevents inappropriate oncological treatment but also ensures timely initiation of disease-specific therapy.
